# The Prognostic Value of Intermedin in Patients with Breast Cancer

**DOI:** 10.1155/2015/862158

**Published:** 2015-01-28

**Authors:** Yi-Min Lu, Jian-Bo Zhong, Hai-Yong Wang, Xiong-Fei Yu, Zhong-Qi Li

**Affiliations:** Department of Oncological Surgery, The First Affiliated Hospital, School of Medicine, Zhejiang University, 79 Qingchun Road, Hangzhou 310003, China

## Abstract

This study aimed to evaluate the prognostic value of preoperative plasma intermedin levels in breast cancer patients. Plasma intermedin levels of 252 breast cancer women and 100 healthy women were determined using radioimmunoassay kit. Adverse event was defined as first local recurrence, distant metastasis, second primary cancer of another organ, or death from any cause during 5-year follow-up. Disease-free survival was defined as the time between surgery and the date of any adverse event whichever appeared first. Overall survival was defined from surgery to death for any cause. The relationships between plasma intermedin levels and clinical outcomes of breast cancer patients were evaluated using multivariate analysis. The results showed that preoperative plasma intermedin levels were substantially higher in patients than in healthy subjects using *t*-test. Intermedin was identified as an independent predictor for 5-year mortality, adverse event, disease-free survival, and overall survival using multivariate analysis. Based on receiver operating characteristic curve analysis, preoperative plasma intermedin levels had high predictive value for 5-year mortality and adverse event. In conclusion, preoperative plasma intermedin levels are highly associated with poor patient outcomes and intermedin may be a potential prognostic biomarker for patients with breast cancer.

## 1. Introduction

Breast cancer (BC) is one of the most common malignancies and the leading cause of mortality in women in China [1]. The incidence of BC is increasing at a surprisingly rapid pace [2]. Traditionally, there is a need for prognostic factors to predict postoperative prognosis after curative resection of the tumor [3]. BC prognostic factors include tumor size, nodal status, histologic grade, histologic type, and hormone receptor status [4, 5]. However, a readily measurable predictive marker predicting BC prognosis would be helpful for early prognostication and risk stratification [6]. Biomarkers are attracting increasing attention as potential predictors of BC patient's survival [7, 8].

Intermedin (IMD), also named as adrenomedullin 2, is a novel member of the calcitonin/calcitonin gene-related peptide (CGRP) family, which includes calcitonin, CGRP, amylin, and adrenomedullin [9, 10]. Among these peptides, calcitonin, CGRP, and amylin have been implicated as mediators of several pathologies such as cardiovascular and renal disorders, sepsis, inflammation, and diabetes [11]; adrenomedullin is expressed in a variety of tumors where it aggravates several of the molecular and physiological features of malignant cells and has been shown to be a mitogenic factor stimulating growth in several cancer types and to encourage a more aggressive tumor phenotype [12–15]. Nowadays, there are a few reports about the investigation of IMD in cancers. However, the effects of IMD generally resemble those of adrenomedullin. IMD participates in a wide range of physiological and pathological events, including cell growth, vasorelaxation, angiogenesis, and apoptosis [16–18]. There is recent evidence that IMD plays a critical role in the vascular remodeling process and tumor angiogenesis and may serve as a novel target for the development of angiogenesis-based anticancer therapies, identifying IMD as a new tumor angiogenic growth factor in a novel way [19]. Up to now, there is a paucity of literature on its relationship with cancer prognosis. This study was designed to verify the association between the preoperative plasma IMD levels and BC patient's survivals.

## 2. Materials and Methods 

### 2.1. Study Population

A total of 252 patients with stages I–III BC were evaluated at The First Affiliated Hospital, School of Medicine, Zhejiang University, Hangzhou, China, from February 2005 to February 2008. None of these patients had other prior systemic diseases like uremia, liver cirrhosis, malignancy, and chronic heart or lung disease. In addition, 100 healthy women were recruited as the control group. This study was permitted by Ethical Committee in The First Affiliated Hospital, School of Medicine, Zhejiang University, and was also conducted according to the Declaration of Helsinki regulations. Written informed consent was obtained from the patients or their relatives when it was difficult to communicate with patients.

### 2.2. Assessment

The collected information in this study included age, menopausal status, hormone receptor status, lymph node status, histologic grade, nuclear grade, tumor-mode-metastasis stage, and tumor size. All patients received radical mastectomy or modified radical mastectomy. Management of all patients was based on international guidelines and adjuvant treatment with radiotherapy, chemotherapy, and hormone therapy was not altered according to the IMD levels.

### 2.3. Follow-Up

After surgery, patients were followed up every 3 months for 3 years and thereafter every 6 months for 2 years. Adverse event was defined as first local recurrence, distant metastasis, second primary cancer of another organ, or death from any cause during follow-up. Disease-free survival (DFS) was defined as the time between surgery and the date of any adverse event whichever appeared first during follow-up. Patients known to be alive with no evidence of adverse event were censored at the last follow-up date. Overall survival (OS) was defined from surgery to death for any cause, and patients who were alive were censored at date of last follow-up visit.

### 2.4. Immunoassay Methods

Peripheral venous blood was obtained from BC patients one day before surgery and from healthy individuals at study entry for IMD measurement. Samples were placed on ice, centrifuged at 3000 g, and plasma-aliquoted and frozen at −70°C. Plasma samples were extracted through a Sep-Pak C18 cartridge (Waters, Milford, MA, USA) following the method of Morimoto et al. [20]. Plasma extracts were assayed in duplicate using IMD radioimmunoassay kit (Phoenix Pharmaceuticals, Belmont, CA, USA). The person carrying the assays was completely blinded to the clinical information.

### 2.5. Statistical Analysis

Statistical analysis of the data was performed using SPSS 19.0 (SPSS Inc., Chicago, IL, USA) and MedCalc 9.6.4.0. (MedCalc Software, Mariakerke, Belgium). The categorical variables were presented as counts (percentage) and the continuous variables were presented as mean ± standard deviation. Intergroup comparisons of the data were performed using Chi-square tests (or Fisher exact tests) for the categorical variables and using *t*-tests for the continuous variables. Multivariate analysis was undertaken through a binary logistic-regression model and multivariate Cox's proportional hazard model in order to evaluate independent predictors of 5-year mortality, adverse event, DFS, and OS with calculated odds ratio (OR), hazard ratios (HR), and 95% confidence interval (CI). Receiver operating characteristic curves were constructed to describe the predictive values with the estimated optimal cut-off points and the calculated areas under curve (AUC). DFS and OS were estimated using the Kaplan-Meier method and the intergroup differences in survival time were tested using the log-rank test. All significant parameters in the univariate analysis were entered into a multivariate model. All *P* values less than 0.05 were considered statistically significant with a 2-tailed test.

## 3. Results

### 3.1. Study Population Characteristics

This study included 252 BC women and 100 healthy women individuals. There was not statistically significant intergroup difference in age. 136 patients (54.0%) had an age of ≥45 y and 116 patients (46.0%) had an age of ≤44 y. 156 patients (61.9%) were premenopausal women and 96 patients (38.1%) were postmenopausal women. 139 patients (55.2%) had tumors of <2 cm in diameter and 113 patients (44.8%) had a tumor of ≥2 cm in diameter. 177 patients (70.2%) had tumor-mode-metastasis stage I or II and 75 patients (29.8%) had tumor-mode-metastasis stage III. 145 patients (57.5%) had negative lymph node status and 107 patients (42.5%) had positive lymph node status. 142 patients (56.4%) had histologic grade I or II and 110 patients (43.6%) had histologic grade III. 138 patients (54.8%) had nuclear grade I or II and 114 patients (45.2%) had nuclear grade III. 143 patients (56.8%) had positive estrogen receptor status and 109 patients (43.2%) had negative estrogen receptor status. 138 patients (54.8%) had positive progesterone receptor status and 114 patients (45.2%) had negative progesterone receptor status.

### 3.2. The Change of Plasma IMD Levels

Plasma IMD levels were statistically significantly higher in the patients than in the controls (166.3 ± 84.1 pg/mL versus 110.9 ± 28.7 pg/mL; *P* < 0.001). In addition, according to some reports about prognostic prediction of other cancers [21, 22], plasma IMD levels were bifurcated at mean value of 166.3 pg/mL. Value of >166.3 pg/mL indicated high IMD level and value of <166.3 pg/mL indicated low IMD level. 102 patients (40.5%) had high IMD level and 150 patients (59.5%) had low IMD level. Six healthy women (6.0%) had high IMD level and 94 healthy women (94.0%) had low IMD level. Using Chi-square test, the difference was statistically significant (*P* < 0.001).

### 3.3. 5-Year Mortality Prediction

**Figure 1:**
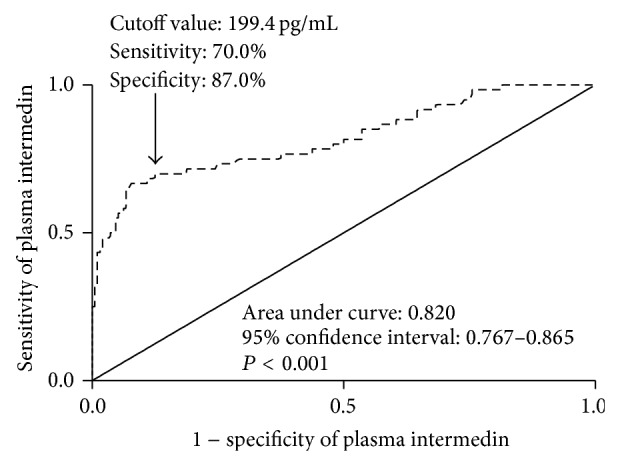
The receiver operating characteristic curve analysis of plasma intermedin levels for 5-year mortality in breast cancer patients. Receiver operating characteristic curves were constructed based on the sensitivity and specificity of plasma intermedin levels for identifying 5-year mortality. Area under curves was calculated based on the receiver operating characteristic curves and expressed as 95% confidence interval. Area under curve ranges from 0.5 to 1.0. An area under curve closer to 1 indicates a higher predictive power.

During 5-year follow-up, 60 patients (23.8%) died.  Table 1 showed that menopausal status, tumor size, tumor-mode-metastasis stage, lymph node status, histologic grade, nuclear grade, estrogen receptor status, progesterone receptor status, and plasma IMD levels were highly associated with mortality of BC women during 5-year follow-up. Multivariate analyses selected high IMD level (OR, 6.321; 95% CI, 3.436–11.627; *P* < 0.001) and positive lymph node status (OR, 2.964; 95% CI, 1.106–5.836; *P* < 0.001) as the independent predictors for 5-year mortality of BC women.  Figure 1 showed that plasma IMD level had high predictive value for 5-year mortality of BC women.

### 3.4. 5-Year Adverse Event Prediction

**Figure 2:**
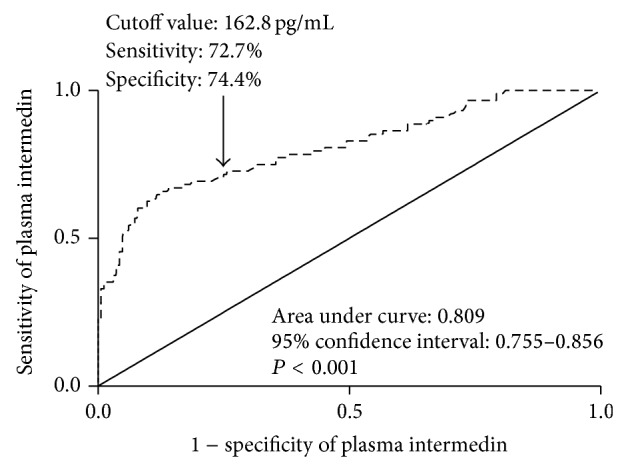
The receiver operating characteristic curve analysis of plasma intermedin levels for 5-year adverse event in breast cancer patients. Receiver operating characteristic curves were constructed based on the sensitivity and specificity of plasma intermedin levels for identifying 5-year adverse event. Area under curves was calculated based on the receiver operating characteristic curves and expressed as 95% confidence interval. Area under curve ranges from 0.5 to 1.0. An area under curve closer to 1 indicates a higher predictive power.

During 5-year follow-up, 88 patients (34.9%) suffered from adverse events.  Table 1 showed that menopausal status, tumor size, tumor-mode-metastasis stage, lymph node status, histologic grade, nuclear grade, estrogen receptor status, progesterone receptor status, and plasma IMD levels were highly associated with adverse events of BC women during 5-year follow-up. Multivariate analyses selected high IMD level (OR, 1.089; 95% CI, 1.062–1.116; *P* < 0.001) and positive lymph node status (OR, 5.691; 95% CI, 2.846–11.379; *P* < 0.001) as the independent predictors for 5-year adverse event of BC women.  Figure 2 showed that plasma IMD level had high predictive value for 5-year adverse event of BC women.

### 3.5. 5-Year OS Analysis

**Figure 3:**
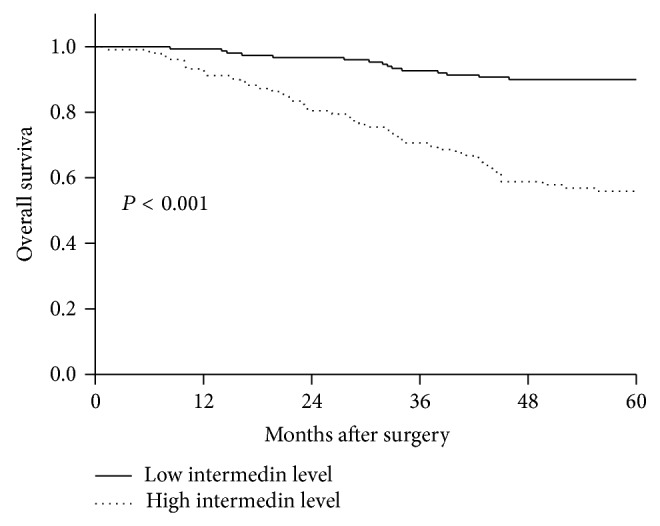
The survival curves for 5-year overall survival in breast cancer patients according to plasma intermedin levels. Plasma intermedin levels were bifurcated at mean value. Value of > mean value indicated high intermedin level and value of < mean value indicated low intermedin level.

During 5-year follow-up, the mean OS time was 52.5 months (95% CI: 50.6–54.3) in all patients.  Table 2 showed that menopausal status, tumor size, tumor-mode-metastasis stage, lymph node status, histologic grade, nuclear grade, estrogen receptor status, progesterone receptor status, and plasma IMD levels were highly associated with 5-year OS of BC women during follow-up. Multivariate analyses selected high IMD level (OR, 4.513; 95% CI, 2.427–8.394; *P* < 0.001) and positive lymph node status (OR, 2.655; 95% CI, 1.042–3.906; *P* < 0.001) as the independent predictors for 5-year OS of BC women. In addition, the mean OS time was 46.0 months (95% CI: 42.4–49.5) in the patients with high IMD level; the mean OS time was 56.9 months (95% CI: 55.2–58.5) in the patients with low IMD level.  Figure 3 showed that women with high IMD level had significantly shorter OS time than those with low IMD level.

### 3.6. 5-Year DFS Analysis

**Figure 4:**
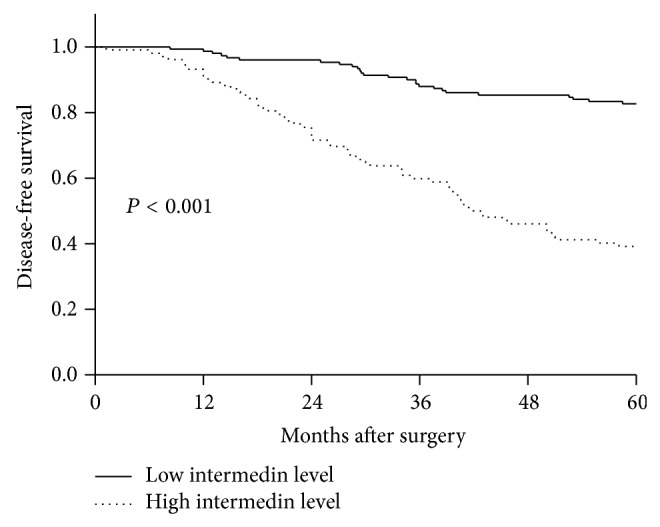
The survival curves for 5-year disease-free survival in breast cancer patients according to plasma intermedin levels. Plasma intermedin levels were bifurcated at mean value. Value of > mean value indicated high intermedin level and value of < mean value indicated low intermedin level.

During 5-year follow-up, the mean DFS time was 49.3 months (95% CI: 47.3–51.4).  Table 2 showed that menopausal status, tumor size, tumor-mode-metastasis stage, lymph node status, histologic grade, nuclear grade, estrogen receptor status, progesterone receptor status, and plasma IMD levels were highly associated with 5-year DFS of BC women during follow-up. Multivariate analyses selected high IMD level (HR, 4.317; 95% CI, 2.355–7.020; *P* < 0.001) and positive lymph node status (HR, 2.428; 95% CI, 1.037–3.733; *P* < 0.001) as the independent predictors for 5-year DFS of BC women. In addition, the mean DFS time was 40.8 months (95% CI: 37.1–44.5) in the patients with high IMD level; the mean DFS time was 55.1 months (95% CI: 53.2–57.0) in the patients with low IMD level.  Figure 4 showed that women with high IMD level had significantly shorter DFS time than those with low IMD level.

## 4. Discussion

To date, to the best of our knowledge, this is the first epidemiologic study to evaluate the prognostic significance of plasma IMD levels in BC patients. The main findings of this study were that plasma IMD levels were also significantly higher in BC patients than in healthy individuals and elevated preoperative plasma IMD levels independently predicted 5-year mortality, adverse event, DFS, and OS in BC women. Thus, elevated plasma IMD levels are associated with BC prognosis independently from other known risk factors for BC, identifying IMD as a potential prognostic biomarker for BC.

Adrenomedullin is an important endocrine and neurocrine integrator of homeostasis in the vascular system, performing diverse important functions in physiogenesis and pathogenesis, including angiogenesis and cancer [23–25]. Similar to adrenomedullin, IMD signals through the calcitonin receptor-like receptor/receptor activity modifying protein complexes and performs vasodilatory and hypotensive actions, with potencies similar to or greater than adrenomedullin [26–28]. The similarities between IMD and adrenomedullin have raised the possibility that IMD may also have a role in angiogenesis and cancer. Recent studies have found that intermedin is overexpressed in hepatocellular carcinomas and adrenal tumors and regulates cell proliferation and survival [29, 30]. In addition, intermedin is identified as a novel regulator for vascular remodeling and tumor vessel normalization by regulating vascular endothelial-cadherin and extracellular signal-regulated kinase, suggesting IMD plays a critical role in the vascular remodeling process and tumor angiogenesis and may serve as a novel target for the development of angiogenesis-based anticancer therapies [18]. The current study found that plasma IMD levels were significantly higher in BC patients compared with healthy individuals. It is proposed that IMD may play some important roles in etiopathogenesis biology of BC.

This study analyzed four prognostic variables including mortality, adverse event, DFS, and OS and showed that plasma IMD level, presented as categorical variable, was identified as an independent prognostic factor. ROC curve showed high predictive value of plasma IMD levels for mortality and adverse event of BC women. In addition, plasma IMD levels were bifurcated at mean value. BC women with high IMD level had significantly shorter OS time and DFS time compared with those with low IMD level. The accumulating evidence substantialized IMD as a potential prognostic biomarker in BC.

The main limitation of our study was that other tumor markers such as cancer antigen 15-3 and cancer antigen 549 were not determined. Thus, the prognostic predictive performances of IMD and other tumor markers cannot be compared based on the receiver operating characteristic curves. However, according to the AUC in this study, IMD has shown the high predictive value for the long-term prognosis of BC patients. Obviously, a larger study including more tumor markers should be performed to investigate the prognostic predictive values of IMD in BC patients.

## 5. Conclusions

This study suggests that high preoperative plasma IMD levels are independently associated with poor patient outcomes and IMD may emerge as a potential prognostic biomarker in BC.

## Figures and Tables

**Table 1:** The factors associated with mortality and adverse events during 5-year follow-up.

Characteristics	Mortality			Adverse event		
	Yes	No	*P* value	Yes	No	*P* value
Number	60	192		88	164	
Age			0.170			0.352
≤44 y	23 (38.3%)	93 (48.4%)		37 (42.0%)	79 (48.2%)	
≥45 y	37 (61.7%)	99 (51.6%)		51 (58.0%)	85 (51.8%)	
Menopausal status			0.030			0.010
Premenopausal	30 (50.0%)	126 (65.6%)		45 (51.1%)	111 (67.7%)	
Postmenopausal	30 (50.0%)	66 (34.4%)		43 (48.9%)	53 (32.3%)	
Tumor size			0.001			0.005
<2 cm	22 (36.7%)	117 (60.9%)		38 (43.2%)	101 (61.6%)	
≥2 cm	38 (63.3%)	75 (39.1%)		50 (56.8%)	63 (38.4%)	
Tumor-mode-metastasis stage			0.003			0.011
I, II	33 (55.0%)	144 (75.0%)		53 (60.2%)	124 (75.6%)	
III	27 (45.0%)	48 (25.0%)		35 (39.8%)	40 (24.4%)	
Lymph node status			<0.001			<0.001
Negative	21 (35.0%)	124 (64.6%)		36 (40.9%)	109 (66.5%)	
Positive	39 (65.0%)	68 (35.4%)		52 (59.1%)	55 (33.5%)	
Histologic grade			<0.001			0.002
I, II	22 (36.7%)	120 (62.5%)		38 (43.2%)	104 (63.4%)	
III	38 (63.3%)	72 (37.5%)		50 (56.8%)	60 (36.6%)	
Nuclear grade			0.001			0.007
I, II	22 (36.7%)	116 (60.4%)		38 (43.2%)	100 (61.0%)	
III	38 (63.3%)	76 (39.6%)		50 (56.8%)	64 (39.0%)	
Estrogen receptor status			<0.001			0.001
Positive	21 (35.0%)	122 (63.5%)		37 (42.1%)	106 (64.6%)	
Negative	39 (65.0%)	70 (36.5%)		51 (57.9%)	58 (35.4%)	
Progesterone receptor status			0.003			0.015
Positive	23 (38.3%)	115 (60.0%)		39 (44.3%)	99 (60.4%)	
Negative	37 (61.7%)	77 (40.0%)		49 (55.7%)	65 (39.6%)	
Intermedin			<0.001			<0.001
Low intermedin level	15 (25.0%)	135 (70.3%)		26 (29.6%)	124 (75.6%)	
High intermedin level	45 (75.0%)	57 (29.7%)		62 (70.4%)	40 (24.4%)	

Variables were presented as counts (percentage). Intergroup comparisons were completed by Chi-square test or Fisher exact test.

**Table 2:** The factors associated with overall survival and disease-free survival during 5-year follow-up.

Characteristics	Overall survival		Disease-free survival	
	Hazard ratio (95% confidence interval)	Univariate analysis *P* values	Hazard ratio (95% confidence interval)	Univariate analysis *P* values
Age (≥45 y versus ≤44 y)	1.431 (0.851–2.409)	0.177	1.240 (0.812–1.894)	0.319
Menopausal status (postmenopausal versus premenopausal)	1.720 (1.037–2.853)	0.036	1.666 (1.097–2.532)	0.017
Tumor size (≥2 cm versus <2 cm)	2.336 (1.381–3.950)	0.002	1.885 (1.236–2.875)	0.003
Tumor-mode-metastasis stage (III versus I, II)	2.112 (1.270–3.514)	0.004	1.787 (1.166–2.741)	0.008
Lymph node status (positive versus negative)	2.817 (1.656–4.790)	<0.001	2.328 (1.521–3.563)	<0.001
Histologic grade (III versus I, II)	2.453 (1.451–4.148)	0.001	2.010 (1.318–3.067)	0.001
Nuclear grade (III versus I, II)	2.277 (1.347–3.851)	0.002	1.853 (1.215–2.827)	0.004
Estrogen receptor status (negative versus positive)	2.695 (1.585–4.582)	<0.001	2.188 (1.432–3.343)	<0.001
Progesterone receptor status (negative versus positive)	2.098 (1.247–3.532)	0.005	1.753 (1.151–2.671)	0.009
Intermedin (high versus low)	5.438 (3.029–9.763)	<0.001	4.885 (3.084–7.739)	<0.001

Univariate Cox's regression analysis was used to calculate the Hazard ratio and 95% confidence interval.
